# ZNF143 Suppresses Cell Apoptosis and Promotes Proliferation in Gastric Cancer via ROS/p53 Axis

**DOI:** 10.1155/2020/5863178

**Published:** 2020-01-28

**Authors:** Yi Zhang, Qing Li, Song Wei, Jing Sun, Xuan Zhang, Ling He, Lu Zhang, Zekuan Xu, Dexuan Chen

**Affiliations:** ^1^Department of General Surgery, The First Affiliated Hospital of Nanjing Medical University, Nanjing, Jiangsu, China; ^2^Department of General Surgery, Jiangsu Province Hospital of Chinese Medicine, Affiliated Hospital of Nanjing University of Chinese Medicine, Nanjing, Jiangsu, China; ^3^School of Medicine, Southeast University, Nanjing, China; ^4^Department of Oncology, The First Affiliated Hospital of Nanjing Medical University, Nanjing, Jiangsu, China; ^5^Jiangsu Key Lab of Cancer Biomarkers, Prevention and Treatment, Jiangsu Collaborative Innovation Center For Cancer Personalized Medicine, School of Public Health, Nanjing Medical University, Nanjing, China

## Abstract

**Aim:**

This study was aimed at identifying the role of zinc finger protein 143 (ZNF143) in gastric cancer (GC) progression.

**Methods:**

The impact of ZNF143 on the proliferation ability and apoptosis of GC cells was detected. The expression of ZNF143 and related targeted genes was determined using Western blot analysis. The reactive oxygen species (ROS) level of GC cells was examined using the ROS generation assay. The role of ZNF143 in the proliferation of GC cells *in vivo* was examined using tumor xenograft assay.

**Results:**

The ectopic overexpression of ZNF143 promoted the proliferation of GC cells, while its knockdown reduced the effect *in vitro*. The downregulation of ZNF143 facilitated cell apoptosis. ZNF143 decreased the ROS level in GC cells, resulting in the reduction of cell apoptosis. Transfection with p53 reversed the antiapoptotic effect of ZNF143, while pifithrin-*α*, a specific inhibitor of p53, reduced the apoptosis in ZNF143-knockdown GC cells. However, p53 had no influence on the ROS level in GC cells. p53 played a key role in inhibiting ROS generation in GC cells, thereby inhibiting apoptosis. The transplanted tumor weight and volume were higher in the ZNF143-overexpressed group than in the ZNF143-knockdown group *in vivo* was examined using tumor xenograft assay.

**Conclusion:**

ZNF143, as a tumor oncogene, promoted the proliferation of GC cells both *in vitro* and *in vivo*, indicating that ZNF143 might function as a novel target for GC therapy.*in vitro*. The downregulation of ZNF143 facilitated cell apoptosis. ZNF143 decreased the ROS level in GC cells, resulting in the reduction of cell apoptosis. Transfection with p53 reversed the antiapoptotic effect of ZNF143, while pifithrin-*in vivo* was examined using tumor xenograft assay.

## 1. Introduction

Gastric cancer (GC) remains one of the most commonly occurring malignancies throughout the world and the fifth commonly diagnosed cancer. The incidence of GC is remarkably elevated in Eastern Asia, including China. It is still the third leading cause of cancer-related mortality worldwide [1, 2]. More than 70% of patients are diagnosed at the advanced stage, and a few patients even lose a chance to undergo surgery. In recent years, continuous researches have been carried out to improve the prognosis of patients with advanced GC. Although considerable improvements have been achieved in understanding developmental mechanisms and therapeutic strategies [2, 3], patients with advanced GC still have poor prognosis. The 5-year overall survival rate of patients with GC remains quite low at approximately 25% [4, 5]. The mechanism of GC progression is still unclear, and effective therapeutic targets to prevent carcinogenic progression are lacking.

Apoptosis plays a pivotal role in the development and progression of malignant tumors, including GC. The evasion of apoptosis is a prominent hallmark of cancer [6]. Dysregulation of the apoptotic signaling pathway facilitates tumor development and accelerates tumor proliferation and metastasis. Most of the cytotoxic anticancer medicines work by inducing apoptosis of cancer cells. Therefore, a comprehensive understanding of the relationship between apoptosis and GC provides a new approach for developing novel therapeutic targets. An in-depth research on the particular molecular mechanism underlying cell apoptosis of GC might help identify novel therapeutic targets for treating GC.

The reactive oxygen species (ROS) plays a vital role in many cellular processes, including autophagy and apoptosis, the two major cell death mechanisms. An increased understanding of the role of ROS shows that ROS are not only metabolic byproducts but also signaling molecules [7, 8]. Excess ROS could activate several injury-producing pathways, such as the nuclear factor-kb (NF-*κ*b) inflammatory and p53 apoptotic pathways [9, 10]. The transcription factor and tumor suppressor p53 is closely related to DNA repair, cellular stress response, and cell cycle control [11, 12]. In response to cellular stress signals, the tumor suppressor p53 activates a large number of genes responsible for cell cycle regulation, DNA repair, and apoptosis [13, 14].

ZNF143, which is a transcription factor, positively regulates many cell cycle-related genes [15]. Functional classification of ZNF143 target genes revealed that many of these genes played an important role in cell proliferation [16]. Izumi et al. demonstrated the role of ZNF143 in tumor growth through transcriptional regulation of DNA replication and cell cycle-associated genes in human prostate cancer PC3 cells [17]. Previous studies reported that ZNF143 enhanced the metastasis of GC by promoting the process of epithelial-mesenchymal transition (EMT) through the PI3K/Akt signaling pathway [18]. However, the role of ZNF143 in GC cell proliferation remains unknown. This study was aimed at identifying the role of ZNF143 during GC progression.

## 2. Results

### 2.1. Expression of ZNF143 Increased (Decreased) after Transfection with ZNF143 Lentivirus (shRNA Lentivirus)

**Figure 1:**
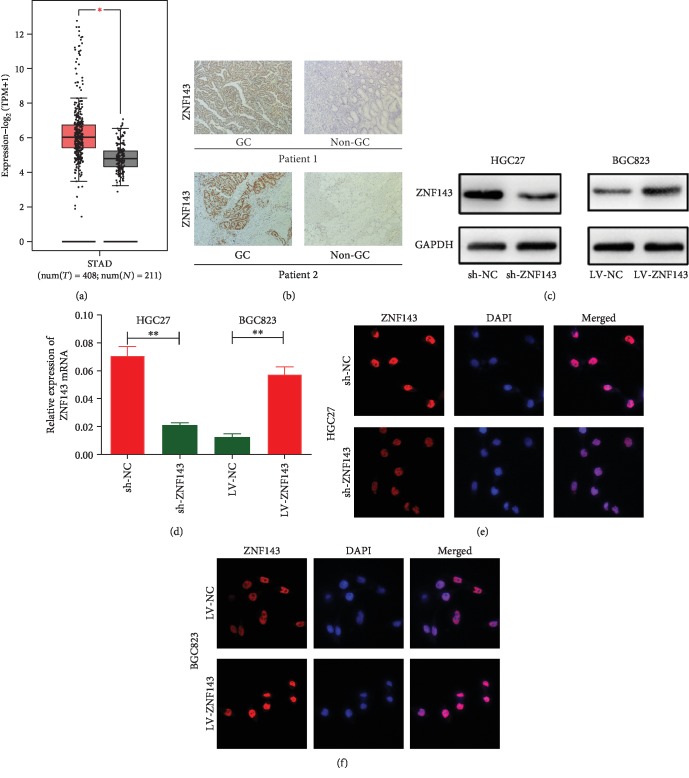
(a) The expression patterns of GC tumors (*n* = 408) and normal GC tissues (*n* = 211) based on The Cancer Genome Atlas (TCGA) and the Genotype-Tissue Expression (GTEx) data in the GEPIA database (http://gepia2.cancer-pku.cn/#analysis). (b) The expression of ZNF143 in GC tumors and corresponding normal tissues using immunohistochemical staining. (c, d) Expression of ZNF143 in HGC27 cells transfected with sh-ZNF143 and in BGC823 cells transfected with LV-ZNF143 lentivirus. (c) The expression of ZNF143 in HGC27 and BGC823 cells analyzed using Western blot analysis. (d) Expression of ZNF143 detected by real-time PCR in HGC27 and BGC823 cells. (e, f) Expression of ZNF143 in HGC27 and BGC823 cells examined using immunofluorescence. ^∗^*P* < 0.05, ^∗∗^*P* < 0.01, and ^∗∗∗^*P* < 0.001. The data were expressed as mean ± standard deviation. GC: gastric cancer; ZNF143: zinc finger protein 143; GAPDH: glyceraldehyde-3-phosphate dehydrogenase; DAPI: 4′,6-diamidino-2-phenylindole dihydrochloride.

A comparison of the expression patterns of ZNF143 in GC tumors (*n* = 408) and normal GC tissues (*n* = 211) based on The Cancer Genome Atlas (TCGA) and the Genotype-Tissue Expression (GTEx) data in the GEPIA database (http://gepia2.cancer-pku.cn/#analysis) revealed that the expression of ZNF143 was higher in GC tumors (Figure 1(a)). Consistently, immunohistochemical staining revealed that the expression of ZNF143 was higher in GC tumors compared with the corresponding normal tissues (Figure 1(b)). HGC27 and BGC823 cell lines were infected with ZNF143 shRNA and ZNF143 lentiviruses, respectively. The Western blot assay and quantitative real-time polymerase chain reaction (PCR) were used to evaluate the transfection efficiency of ZNF143 in GC cells. Figures 1(c) and 1(d) show that the expression of ZNF143 decreased in HGC27 cells transfected with shRNA lentivirus compared with the negative control, and it was overexpressed in BGC823 cells transfected with ZNF143 lentivirus. The transfection efficiency was also evaluated using immunofluorescence confocal microscopy, which was consistent with the results of Western blot assay and quantitative real-time PCR (Figures 1(e) and 1(f)).

### 2.2. ZNF143 Promoted the Proliferation of GC Cells

**Figure 2:**
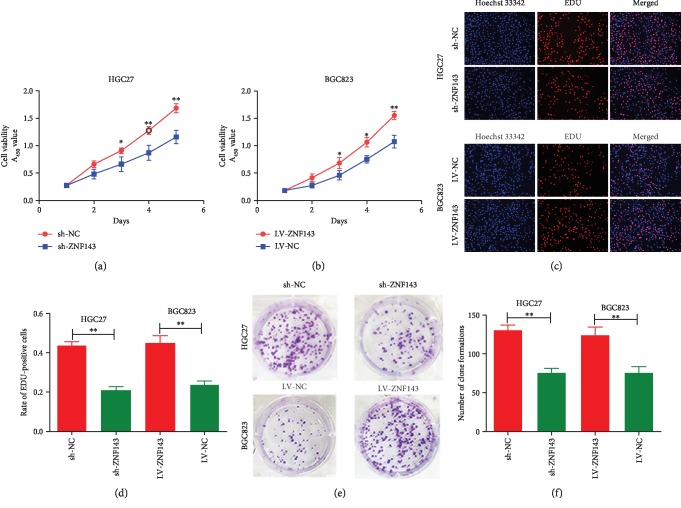
ZNF143 promoted the proliferation of GC cells *in vitro*. (a, b) Effects of ZNF143 on the proliferation ability of GC cells explored using the CCK-8 assay. (c) ZNF143 knockdown inhibited the proliferation of HGC27 cells, and overexpression of ZNF143 promoted the proliferation of BGC823 cells, as detected using EDU assays. (d) Rate of EDU-positive cells. (e) Colony formation assays showing that ZNF143 knockdown inhibited the proliferation of HGC27 cells, while the overexpression of ZNF143 promoted the proliferation of BGC823 cells. (f) Number of clones formed. ^∗^*P* < 0.05, ^∗∗^*P* < 0.01, and ^∗∗∗^*P* < 0.001. The data were expressed as mean ± standard deviation. CCK8: Cell Counting Kit-8; EDU: 5-ethynyl-2′-deoxyuridine.

The Cell Counting Kit-8 (CCK-8) assay was employed to examine the impact of ZNF143 on the proliferation ability of GC cells. The proliferation ability of HGC27 cells transfected with sh-ZNF143 lentivirus was inhibited compared with that of scrambled shRNA (Figure 2(a)). The difference was statistically significant (*P* < 0.05). On the contrary, the overexpression of ZNF143 promoted the growth of BGC823 cells transfected with ZNF143 lentivirus (Figure 2(b)). Figures 2(c) and 2(d) show that the percentage of EDU-positive cells decreased in HGC27 cells transfected with the sh-ZNF143 lentivirus compared with the negative control, whereas the BGC823 cells transfected with LV-ZNF143 lentivirus showed the opposite effects. The colony formation assay was also used to explore the effect of ZNF143 on the proliferation ability of GC cells. Figures 2(e) and 2(f) show that the HGC27 cells transfected with sh-ZNF143 lentivirus showed fewer clones compared with the control, while BGC823 cells transfected with LV-ZNF143 lentivirus showed more clones compared with the negative control. Taken together, the findings revealed that ZNF143 promoted the proliferation of GC cells.

### 2.3. ZNF143 Inhibited Cell Cycle Arrest of the G1 Phase and Suppressed the Apoptosis of GC Cells

**Figure 3:**
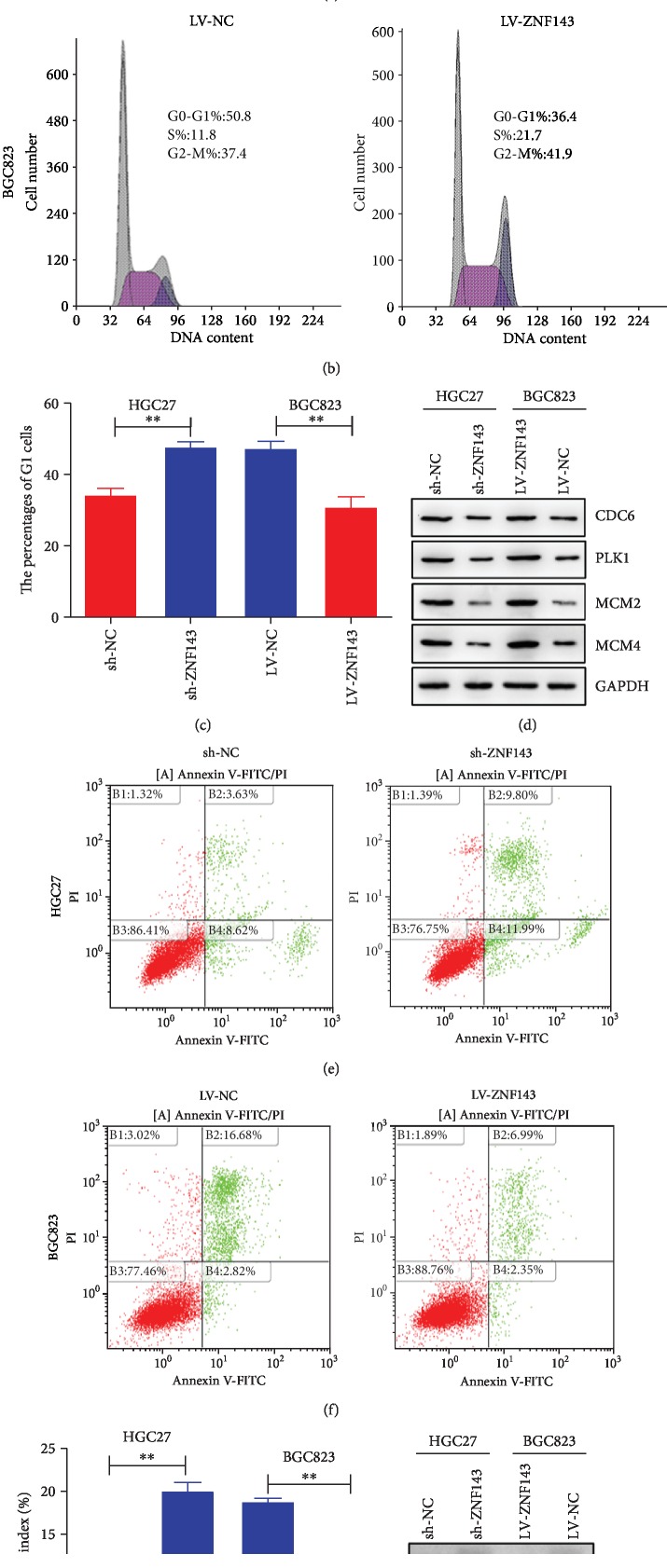
ZNF143 inhibited cell cycle arrest in the G1 phase and the apoptosis of GC cells. (a, b) Cell cycle distribution of GC cells measured using flow cytometry. (c) Percentage of G1 phase. (d) Protein expression of CDC6, PLK1, MCM2, and MCM4 detected using Western blot analysis. (e, f) Apoptotic rate of GC cells measured using flow cytometry. (g) Apoptotic rate of GC cells. (h) Protein expression levels of p53, Bcl-2, Bax-cl, and Bax detected using Western blot analysis. ^∗^*P* < 0.05, ^∗∗^*P* < 0.01, and ^∗∗∗^*P* < 0.001. The data were expressed as mean ± standard deviation. CDC6: cell division cycle 6; PLK1: polo-like kinase 1; MCM2: minichromosome maintenance complex component 2; MCM4: minichromosome maintenance complex component 4; p53: tumor protein 53; Bcl-2: B-cell lymphoma-2; Bcl-xl: BCL2-like 1; Bax: BCL2 associated X; GAPDH: glyceraldehyde-3-phosphate dehydrogenase.

The cell cycle distribution of GC cells was measured using fluorescence-activated cell sorting (FACS) to explore the exact mechanism underlying the effect of ZNF143 on the proliferation ability of GC cells. The results revealed that ZNF143 knockdown could induce the cell cycle arrest in the G1 phase in HGC27 cells transfected with sh-ZNF143 lentivirus. However, the overexpression of ZNF143 reduced the cell cycle arrest in the G1 phase in BGC823 cells (Figures 3(a)–3(c)). The expression of cell cycle*-*related genes in GC cells was also detected using the Western blot assay. The results showed that ZNF143 knockdown downregulated the expression of cell division cycle 6 homolog (CDC6), polo-like kinase 1 (PLK1), minichromosome maintenance complex component 2 (MCM2), and MCM4, while the overexpression of ZNF143 showed the opposite effects (Figure 3(d)). Besides, the effect of ZNF143 on the apoptosis of GC was explored using flow cytometry. ZNF143 knockdown was found to promote the apoptosis of HGC27 cells, while the overexpression of ZNF143 inhibited the apoptosis of BGC823 cells transfected with ZNF143 lentivirus compared with the control (Figures 3(e)–3(g)). The expression of some apoptosis-related genes, such as p53, Bcl-2, and Bax, was also detected. The results of Western blot analysis showed that ZNF143 knockdown decreased the expression of antiapoptotic proteins Bcl-2 and Bcl-xl and increased the expression of tumor suppressor gene p53 and Bax (Figure 3(h)). In general, the findings revealed that ZNF143 could reduce cell cycle arrest in the G1 phase and suppress apoptosis in GC cells.

### 2.4. ZNF143 Reduced p53-Dependent ROS-Mediated Apoptosis in GC Cells

The findings revealed that the expression of apoptotic regulatory genes p53, Bax, Bcl-2, and Bcl-xl was modulated by ZNF143. p53, as a proapoptotic protein, played a pivotal role in the cell apoptosis and cell cycle processes. Given that ROS also participated in the apoptosis extensively and was associated with p53-dependent cell cycle arrest, it was presumed that interactions between p53 and ROS signaling transduction pathways might exist. As previously described, the overexpression of ZNF143 could downregulate the expression of p53. p53 is associated with ROS generation, which is an important mechanism of cell apoptosis. Next, this study explored the relationship between p53 and ROS and its role in the apoptosis of GC cells.

**Figure 4:**
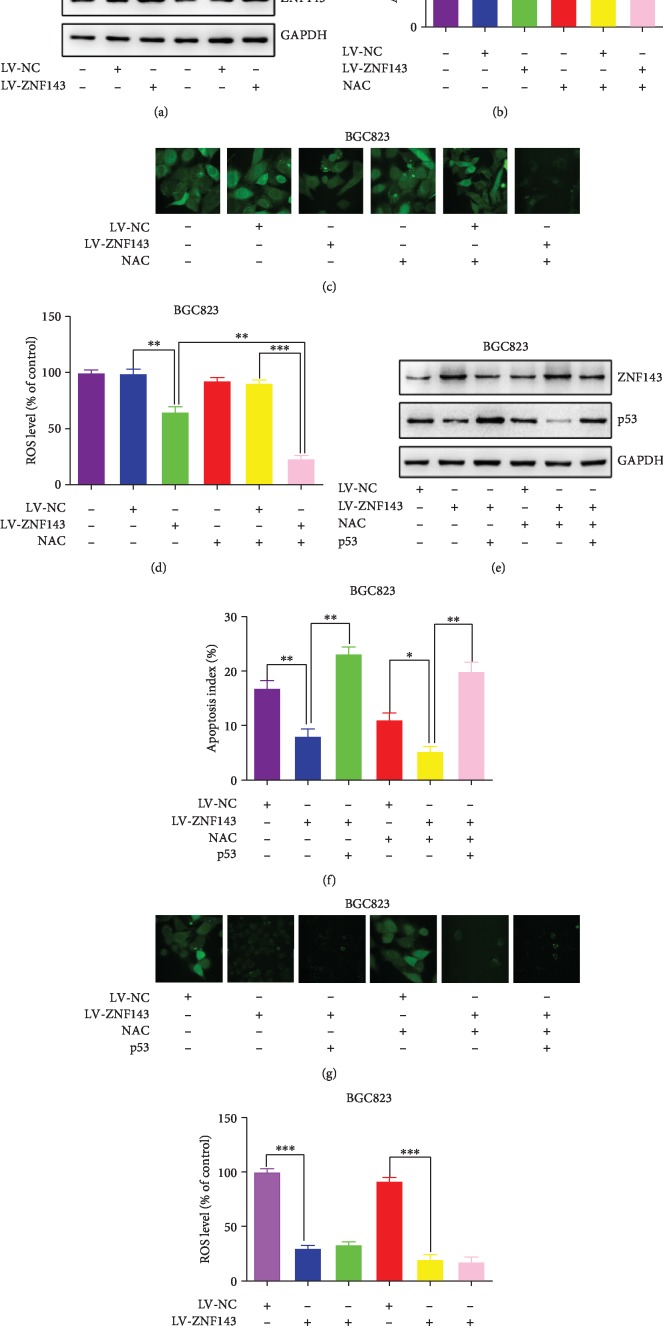
Overexpression of ZNF143 inhibited p53-dependent ROS-mediated apoptosis in BGC823. (a) Protein level of ZNF143 detected using Western blot assay. (b) Apoptotic rate of GC cells measured using flow cytometry. (c, d) ROS level of GC cells detected using confocal microscopy. (e) Expression of ZNF143 and p53 in GC cells analyzed using Western blot assay. (f) Apoptotic rate of GC cells measured using flow cytometry. (g, h) ROS level of GC cells after different treatments were detected using confocal microscopy. ^∗^*P* < 0.05, ^∗∗^*P* < 0.01, and ^∗∗∗^*P* < 0.001. The data were expressed as mean ± standard deviation. NAC: N-acetylcysteine; ROS, reactive oxygen species.

The expression of ZNF143 in BGC823 cells was detected once again to verify the infection efficiency. The result showed that the expression of ZNF143 increased in ZNF143 lentivirus*-*infected cells compared with the controls (Figure 4(a)). Consequently, the overexpression of ZNF143 suppressed the apoptosis of BGC823 cells. In addition, the ROS generation inhibitor N-acetylcysteine (NAC), used as a positive control, also suppressed the apoptosis of BGC823 cells (Figure 4(b)). The effects of ZNF143 on ROS generation are shown in Figures 4(c) and 4(d). This study found that the overexpression of ZNF143 significantly inhibited the ROS generation in BGC823 cells. In addition, the overexpression of ZNF143 decreased the expression of p53, and the inhibitor of ROS generation, NAC, enhanced the effect of ZNF143 on the expression of p53 (Figure 4(e)). The results of flow cytometry in Figure 4(f) showed that ZNF143 exerted antiapoptotic effects while NAC reinforced its role in BGC823 cells infected with ZNF143 lentivirus. Furthermore, the wild-type p53 was transfected into BGC823 cells to rescue the expression of p53. The result showed that the tumor suppressor gene p53 reversed the antiapoptotic effects of ZNF143, whereas p53 had no influence on the ROS generation (Figures 4(g) and 4(h)). The results revealed that the overexpression of ZNF143 inhibited the ROS generation and then suppressed cell apoptosis by downregulating the expression of p53 in BGC823 cells.

**Figure 5:**
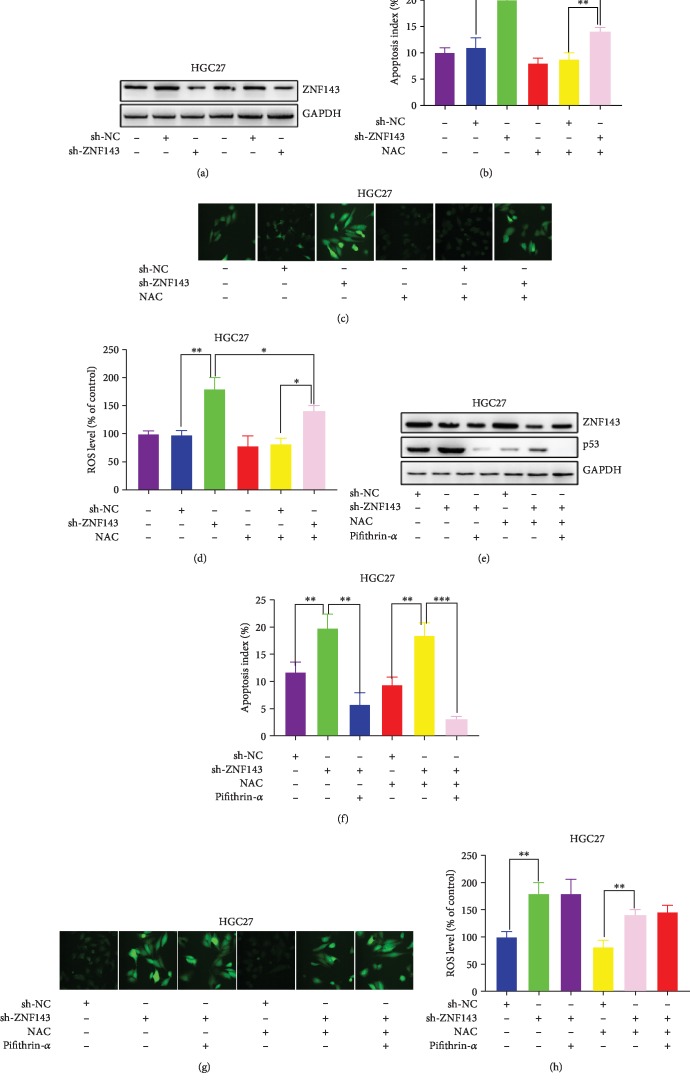
Knockdown of ZNF143 could promote p53-dependent ROS-mediated apoptosis in HGC27. (a) Protein level of ZNF143 detected using Western blot analysis. (b) Apoptotic rate of GC cells measured using flow cytometry. (c, d) ROS level of GC cells detected using confocal microscopy. (e) Expression of ZNF143 and p53 in GC cells analyzed using Western blot assay. (f) Apoptotic rate of GC cells measured using flow cytometry. (g, h) ROS level of GC cells after different treatments were detected using confocal microscopy. ^∗^*P* < 0.05, ^∗∗^*P* < 0.01, and ^∗∗∗^*P* < 0.001. The data were expressed as mean ± standard deviation.

Further, the expression of ZNF143 decreased in HGC27 cells transfected with sh-ZNF143 (Figure 5(a)). The knockdown of ZNF143 facilitated the apoptosis of GC cells, while NAC attenuated the effect (Figure 5(b)). The effects of ZNF143 on ROS generation are shown in Figures 5(c) and 5(d). The downregulation of ZNF143 significantly increased the ROS level of HGC27 cells, and NAC suppressed the ROS generation in HGC27 cells.  Figure 5(e) shows that the downregulation of ZNF143 increased the expression of p53, and treatment with NAC blocked the upregulation of p53. Moreover, the results of flow cytometry showed that the downregulation of ZNF143 promoted HGC27 cell apoptosis and the ROS generation inhibitor NAC inhibited the apoptosis of HGC27 cells transfected with sh-ZNF143 lentivirus (Figure 5(f)). In the rescue experiment, the p53-specific inhibitor, pifithrin-*α*, was employed to inhibit the p53 protein. The results indicated that the blockage of p53 could prevent cell apoptosis in the ZNF143-knockdown HGC27 cells (Figure 5(f)). However, pifithrin-*α* had no influence on the ROS generation (Figures 5(g) and 5(h)).

In general, these data revealed that ZNF143 could reduce p53-dependent ROS-mediated apoptosis in GC cells, resulting in a decreased apoptotic rate in GC cells.

### 2.5. ZNF143 Promoted the GC Tumorigenesis of Nude Mice *In Vivo*

**Figure 6:**
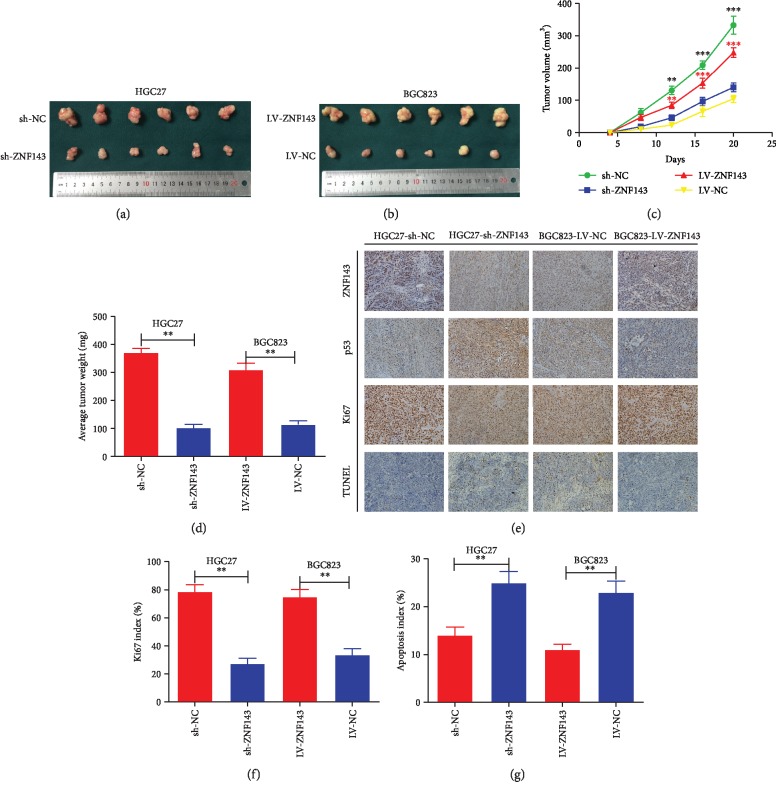
ZNF143 promoted xenograft tumor growth of GC cells in nude mice. (a, b) Tumors were obtained from nude mice injected subcutaneously with GC cells. (c) Growth curve of tumor volumes. (d) Average tumor weight of nude mice. (e) Representative images of ZNF143, p53, Ki67, and TUNEL staining in the xenografts. Original magnification, 200x. (f) Percentages of cells positive for Ki67 staining presented as a Ki67 index. (g) Percentages of TUNEL-positive cells presented as an apoptosis index. ^∗^*P* < 0.05, ^∗∗^*P* < 0.01, and ^∗∗∗^*P* < 0.001. The data were expressed as mean ± standard deviation. Ki67: nuclear-associated antigen; TUNEL: terminal deoxynucleotidyl transferase-mediated dUTP-biotin nick end labeling assay.

HGC27 and BGC823 cells were injected into nude mice to explore the effect of ZNF143 on tumorigenicity in vivo. The size and volume of tumors were calculated every 4 days. After 4 weeks, the nude mice were sacrificed, the transplanted tumors were harvested, and the weight was measured. The volume and weight of nude mice injected with HGC27 cells infected with sh-ZNF143 lentivirus were found to be significantly smaller than the volume and weight of those injected with HGC27 cells infected with the negative control. In addition, the nude mice injected with BGC823 cells infected with ZNF143 lentivirus showed the opposite results (Figures 6(a)–6(d)). Further, the expression of ZNF143 protein in nude mice was examined by immunochemical assay. Compared with the negative control, ZNF143 protein in the ZNF143-knockdown group showed lower expression, while its expression significantly increased in the ZNF143-overexpressed group. The results of immunochemistry assay showed that the expression of p53 was higher in the ZNF143-knockdown group, while opposite results were obtained in the ZNF143-overexpressed group (Figure 6(e)). Ki67 staining was used to determine the proliferative ability of GC cells. The results revealed that Ki67 was strongly expressed in the ZNF143-overexpressed group, while the results in the ZNF143-knockdown group were opposite (Figure 6(f)). In addition, TUNEL assays were performed to detect the apoptosis of GC cells. The apoptotic rate of HGC27 cells transfected with sh-ZNF143 lentivirus was found to be remarkably higher compared with that of the negative control. However, the apoptotic rate of BGC823 cells transfected with ZNF143 lentivirus showed opposite results (Figure 6(g)). In general, the results revealed that ZNF143 promoted the proliferation and inhibited the apoptosis of GC cells in nude mice.

## 3. Discussion

GC continues to be a highly lethal malignancy in spite of using varied treatment methods [19, 20]. Hence, a better understanding of the mechanisms of GC proliferation is necessary [21]. This study suggested that ZNF143, a zinc finger transcription factor, promoted the proliferation and suppressed the apoptosis via the ROS/p53 axis in GC. First, the expression of ZNF143 was found to be higher in GC tumors using the TCGA and GTEx data. Immunohistochemical staining also confirmed that the expression of ZNF143 was higher in GC tumors compared with the corresponding normal tissues. This study provided evidence that stable expression of ZNF143 greatly promoted the proliferation of GC cells and upregulated the expression of cell cycle-related genes. Further, ZNF143 overexpression suppressed the apoptosis of GC cells *in vitro* and promoted tumor growth in nude mice *in vivo*. This was the first study to reveal the proliferative role of ZNF143 in GC cells.

ZNF143 is a human homolog of *Xenopus* transcriptional activator staf associated with selenocystyl tRNA transcription [22]. Anno et al. demonstrated that ZNF143 exhibited an inherently bidirectional transcription activity and controlled the expression of divergent protein-protein and protein-noncoding RNA gene pairs [23]. Myslinski et al. reported that 1175 ZNF143-binding sites were distributed in 938 promoters of mammalian protein genes, indicating that ZNF143 was involved in the transcriptional regulation of a large number of protein-coding genes [16]. It also has been reported that ZNF143 may be an important adaptive mechanism for cell survival and drug resistance in mitochondrial respiratory dysfunction [24].

Excessive ROS production can suppress the transcription of many genes associated with cellular growth and mitochondrial functions and induce the upregulation of p53 and p21 [25]. ROS are generated by the normal energy metabolism of the cell. Evidence revealed that ROS functioned as important cell signaling molecules [26]. However, excessive accumulation of ROS owing to the presence of inducer-like arsenic could increase lipid peroxidation (LPO) and DNA damage [27]. Apoptosis is an important cause of cell proliferation inhibition. Convincing evidence shows that excessive ROS production exceeds cellular antioxidant defenses, triggering apoptosis. The p53 gene is known as the guardian of the genome and is capable of activating cell cycle, DNA repair, and apoptosis to maintain the stability of the cell genome [28]. Bcl-2 and Bax are very important to apoptosis. The Bcl-2 gene has an antiapoptotic effect, while the Bax is known for its proapoptotic effect [29, 30]. Cell cycle arrest is another important reason for growth inhibition [31]. Many anticancer agents inhibited malignant growth by arresting the cell cycle at the G1 phase [32]. Many ZNF143 target genes were related to cell cycle and DNA replication, such as CDC6, PLK1, and MCM DNA replication proteins [17]. Among the ZNF143-targeted cell cycle-associated kinases, both PLK1 and Aurora kinase B (AUPKB) were overexpressed in a large variety of cancers [33]. The MCM proteins were required for prereplication complex formation, DNA replication initiation, and DNA synthesis [34]. In this study, ZNF143 was found to regulate the cell cycle-related genes, such as PLK1, CDC6, MCM2, and MCM4, and apoptosis-related genes, such as p53, Bcl-xl, Bcl-2, and Bax. The overexpression of ZNF143 suppressed the apoptosis via the ROS/p53 axis in GC, suggesting that ZNF143 promoted the proliferation and suppressed the apoptosis of GC cells. Moreover, the exact mechanism of how ZNF143 promoted the proliferation of GC remains to be explored in further studies.

## 4. Materials and Methods

### 4.1. GC Cell Lines and Specimens

HGC27 and BGC823 cell lines were purchased from the Cell Center of Shanghai Institutes for Biological Sciences (Shanghai, China) and cultured in the Roswell Park Memorial Institute-1640 (RPMI-1640) medium supplemented with 10% fetal bovine serum (FBS) at 37°C in a humidified atmosphere with 5% CO_2_. Human GC specimens and adjacent normal tissues were collected from patients who underwent surgery at the Department of General Surgery, the First Affiliated Hospital of Nanjing Medical University. The study protocol was reviewed and approved by the Ethics Committee of the First Affiliated Hospital of Nanjing Medical University.

### 4.2. Western Blot Analysis

The protein was extracted from GC cells and transferred to polyvinylidene difluoride membranes. The transferred membranes were then blocked with 5% nonfat powdered milk for 2 h and incubated with primary antibodies overnight at 4°C. The membranes were then incubated for 2 h with secondary antibodies at room temperature. The primary antibodies used in this study were as follows: ZNF143 (Santa Cruz Biotechnology, Texas, USA; 1 : 200 dilution), CDC6, PLK1, MCM2, MCM4, p53, Bcl-2, Bcl-xl, Bax, and GAPDH (Cell Signaling Technology, MA, USA; 1 : 1000 dilution). GAPDH was used as an internal control.

### 4.3. Quantitative Real-Time PCR

The total RNA was extracted from GC cells using TRIzol (Takara, Shiga, Japan) according to the manufacturer's protocols, and cDNA was synthesized using PrimeScript RT Reagent (Takara, Shiga, Japan). The PCR reactions were performed using a 7500 real-time PCR system (Applied Biosystems, CA, USA). The gene expression levels were determined by the *ΔΔ*CT method, with the expression of ZNF143 normalized to the expression of *β*-actin. The primers were as follows: ZNF143: forward 5′-AGACTTGGCAGCATTCCATAC-3′, reverse 5′-CCATTGGATGTTGCTACTAAGGT-3′, and *β*-actin: forward 5′-GCATCGTCACCAACTGGGAC-3′, reverse 5′-ACCTGGCCGTCAGGCAGCTC-3′.

### 4.4. Immunofluorescence

The cells were seeded into 24-well plates for 24 h, followed by washing with phosphate-buffered saline (pH = 7.4). After the cells were fixed with ice-cold methanol for 15 min and blocked with 10% goat serum containing 0.05% Tween-20 for 1 h, they were incubated overnight at 4°C with primary antibody against ZNF143 (Santa Cruz Biotechnology, CA, USA; 1 : 200 dilution). The cells were then washed and incubated with Alexa Fluor 594 (Invitrogen, CA, USA; 1 : 1000 dilution) for 2 h at room temperature. Then, they were washed and treated with 4′,6-diamidino-2-phenylindole (DAPI; 10 ng/mL) for 5 min. Immunostaining was observed using a confocal laser scanning microscope (FluoView FV10i, Olympus, Japan).

### 4.5. CCK-8 Assay

Two thousand cells were seeded in 96-well plates and cultured in RPMI-1640 medium supplemented with 10% FBS for 6 days. The cell proliferation ability was examined using CCK-8. In brief, 10 *μ*L of CCK-8 solution was added to each plate. After incubating for 2 h in 37°C, the cell viability was revealed by the absorbance measured at 450 nm.

### 4.6. EDU Incorporation Assay

The proliferation ability of GC cells was examined using the EDU assay kit (RiboBio, Guangzhou, China). In brief, HGC27-ZNF143-shRNA, HGC27-ZNF143-sh-NC, BGC823-LV-NC, and BGC823-LV-ZNF143 stable cells were seeded into 96-well plates at a density of 5 × 10^3^ cells per well and cultured for 24 h. After incubating with 10 *μ*M of EDU for 2 h, the GC cells were fixed in 4% paraformaldehyde for 15 min and permeabilized with 0.3% Triton X-100 at 37°C for 20 min. After washing with phosphate-buffered saline (PBS), the cells were stained with 100 *μ*L of 1x ApolloR reaction cocktail for 30 min. Afterward, 100 *μ*L of Hoechst 33342 (5 *μ*L/mL) was used to stain the cellular nuclei for 20 min, and the number of EDU-positive cells was counted in five random fields using a fluorescent microscope.

### 4.7. Colony Formation Assay

Five hundred cells were plated in six-well plates and cultured in RPMI-1640 medium containing 10% FBS. When the colony was obvious after 9 days, the plates were washed with PBS and stained with Giemsa for 15 min. Colonies consisting of 50 cells or more were counted and photographed for statistical analysis. All experiments were performed in triplicate.

### 4.8. Vector Constructs, Lentivirus Production, and Cell Transfection

Vectors for the overexpression and shRNA targeting of ZNF143 using lentiviral gene transfer containing the puromycin resistance sequence were constructed by GenePharma Biotech (Shanghai, China). The scrambled lentiviral construct was used as a negative control. BGC823 and HGC27 cells were infected with the lentiviral vectors. The LV-ZNF143, LV-NC, and shRNA-ZNF143 lentiviral vectors were used at an appropriate multiplicity of infection to infect BGC823 and HGC27 cells grown to 40%–50% confluence. Stable cell lines (BGC823-LV-ZNF143, BGC823-LV-NC, HGC27-ZNF143-shRNA, and HGC27-ZNF143-sh-NC) were generated by selecting transfected cells in culture mediums containing 5 *μ*g/mL puromycin (Sigma-Aldrich, MO, USA) for 5 days. The expression of ZNF143 was then analyzed using quantitative reverse transcriptase- (qRT-) PCR and Western blot analysis. Human wild-type p53 (RiboBio, Guangzhou, China) was transiently transfected into cells using Lipofectamine 2000 (Life Technologies, CA, USA) following the manufacturer's protocol.

### 4.9. Cell Cycle Assay

For cell cycle analysis, GC cells were harvested after 48 h incubation. Before flow cytometry (FCM) detection, the cells were washed twice with PBS, incubated with RNase, and stained with Propidium Iodide (PI) staining solution (500 *μ*L) for 15 min at room temperature for cell cycle analysis. The FACSCalibur flow cytometry with Cell Quest software (Becton Dickinson, NJ, USA) was used to analyze the DNA content of labeled cells.

### 4.10. Apoptosis Analysis Using Flow Cytometry

A total of 5 × 10^5^ GC cells in six-well plates were cultured in RPMI-1640 medium containing 10% FBS for 24 h. After the GC cells were harvested, washed, and resuspended in ice-cold PBS, they were stained with PI (10 *μ*g/mL; Sigma) and Annexin V-FITC (50 *μ*g/mL, Becton Dickinson, NJ, USA) and incubated for 15 min at 37°C. The data were analyzed using a FACSCalibur flow cytometer (Becton Dickinson, NJ, USA) with the Cell Quest software.

### 4.11. Detection of Intracellular ROS Level

The production of peroxides was measured using the ROS assay kit (Beyotime, Haimen, China). Stable cell lines (20,000 cells/well) cultured on collagen-coated glass coverslips were incubated with 10 *μ*M 2,7-dichlorodihydrofluorescein diacetate (DCFH-DA) in a serum-free medium at 37°C for 20 min. Subsequently, the cells were imaged with a laser scanning confocal microscope (Nikon, Japan) at excitation and emission wavelengths of 488 and 525 nm, respectively. The fluorescence intensity was regarded as the generation of ROS. The cells with NAC (10 mM) were preincubated for 2 h before detecting the level of intracellular ROS to see whether inhibition of apoptosis in the BGC823-LV-ZNF143 cell lines was induced by NAC.

### 4.12. Immunohistochemistry

The immunochemical analysis was performed as described in a previous study [15]. ZNF143 (diluted 1 : 200; Abcam, Cambridge, UK), p53, and Ki67 (diluted 1 : 200; Cell Signal Technology, MA, USA) were used as primary antibodies.

### 4.13. TUNEL Assay

The cancer tissues of nude mice were fixed in 4% formalin and embedded in paraffin. Then, the cell apoptosis was examined using a TUNEL apoptosis detection kit (KeyGen, Nanjing, China). The apoptotic cells were captured and counted in five random fields (magnification, ×200) for each group. The apoptotic index of the cancer cells = apoptotic cells/total cells × 100%.

### 4.14. Tumor Xenografts in Animals

The BALB/c female nude mice (4 weeks old) were purchased from the Animal Center of Nanjing Medical University. The GC cells were subcutaneously injected with 2 × 10^6^ cells resuspended in 200 *μ*L of PBS into the flank of nude mice. The care of experimental animals was conducted according to the guidelines of the Nanjing Medical University Institutional Animal Care and Use Committee. The tumor size was evaluated with calipers every 4 days. The volume of the tumor was calculated using the following formula: tumor volume = (length × width^2^) × 0.5.

### 4.15. Statistical Analysis

All data were expressed as mean ± standard deviation. The statistical analyses were performed using two-tailed Student *t*-test with the Statistical Product and Service Solutions software. Categorical data were evaluated using the *χ*^2^ test. A *P* value < 0.05 was considered statistically significant.

## 5. Conclusions

The findings of this study suggested that ZNF143 could induce cell cycle arrest by targeting cell cycle-related genes. The results also revealed that ZNF143 could induce p53-dependent ROS-mediated apoptosis in GC cells. The findings provided a novel insight into the role of ZNF143 in GC, suggesting that ZNF143 might be a novel target for anticancer therapy. Moreover, the exact mechanisms of how ZNF143 regulated the proliferation in GC still need to be further examined.

## Data Availability

The data used to support the findings of this study are available from the corresponding authors upon request.
